# Target of obstructive sleep apnea syndrome merge lung cancer: based on big data platform

**DOI:** 10.18632/oncotarget.15372

**Published:** 2017-02-16

**Authors:** Lifeng Li, Jingli Lu, Wenhua Xue, Liping Wang, Yunkai Zhai, Zhirui Fan, Ge Wu, Feifei Fan, Jieyao Li, Chaoqi Zhang, Yi Zhang, Jie Zhao

**Affiliations:** ^1^ Biotherapy Center, The First Affiliated Hospital of Zhengzhou University, Zhengzhou 450052, Henan, China; ^2^ Department of Oncology, The First Affiliated Hospital of Zhengzhou University, Zhengzhou 450052, Henan, China; ^3^ Department of Pharmacy, The First Affiliated Hospital of Zhengzhou University, Zhengzhou 450052, Henan, China; ^4^ Engineering Research Center of Digital Medicine, Zhengzhou 450052, Henan, China; ^5^ Engineering Laboratory for Digital Telemedicine Service, Zhengzhou 450052, Henan, China; ^6^ Department of Respiratoty and Sleep Disease, The First Affiliated Hospital of Zhengzhou University, Zhengzhou 450052, Henan, China

**Keywords:** obstructive sleep apnea syndrome, lung cancer, big data platform

## Abstract

Based on our hospital database, the incidence of lung cancer diagnoses was similar in obstructive sleep apnea Syndrome (OSAS) and hospital general population; among individual with a diagnosis of lung cancer, the presence of OSAS was associated with an increased risk for mortality. In the gene expression and network-level information, we revealed significant alterations of molecules related to HIF1 and metabolic pathways in the hypoxic-conditioned lung cancer cells. We also observed that GBE1 and HK2 are downstream of HIF1 pathway important in hypoxia-conditioned lung cancer cell. Furthermore, we used publicly available datasets to validate that the late-stage lung adenocarcinoma patients showed higher expression HK2 and GBE1 than early-stage ones. In terms of prognostic features, a survival analysis revealed that the high GBE1 and HK2 expression group exhibited poorer survival in lung adenocarcinoma patients. By analyzing and integrating multiple datasets, we identify molecular convergence between hypoxia and lung cancer that reflects their clinical profiles and reveals molecular pathways involved in hypoxic-induced lung cancer progression. In conclusion, we show that OSAS severity appears to increase the risk of lung cancer mortality.

## INTRODUCTION

Epidemiological evidence has suggested that obstructive sleep apnea Syndrome (OSAS) is associated with a higher prevalence of cancer and cancer-related mortality; laboratory-based observations have also revealed that constitutive components of OSAS are mechanistically involved in accelerated tumor growth and progression. However, little information is available on the association between OSAS and lung cancer. To clarify this possibility, we utilized a hospital-based database to examine whether the presence of OSAS increased lung cancer incidence and risk of progression or mortality from cancer. Next, we performed gene microarray analyses of datasets from the Cancer Genome Atlas (TCGA) and Gene Expression Omnibus (GEO) to evaluate underlying molecular mechanisms that involved in clinic pathological and prognostic features of patients with lung cancer.

OSAS is a highly prevalent chronic disease, characterized by repetitive episodes of upper-airway obstruction during sleep. Population prevalence estimates range between 6% and 17%, and many individuals with this disease remain undiagnosed [1]. Extensive studies demonstrate that OSAS contributes to developing many diseases such as cardiovascular and metabolic diseases, behavioral and cognitive dysfunction, as well as affects the quality of life [2–6].

In recent years, several cohort studies unravel potential associations between OSAS and cancers, suggesting that patients with OSAS increase risks for developing solid tumors and promoting adverse cancer outcomes [7]. Results from Wisconsin Cohort study indicated that severe sleep disordered breathing was associated with an almost five-fold risk of cancer death [8]. Similarly, another study in women suggested that the risk of breast cancer was increased in OSAS patients [9]. Consistent with these reports from the cohorts’ studies, the evidence from laboratory and animal experiments demonstrated that intermittent hypoxia and sleep fragmentation, as two major components of OSAS, accelerated tumor growth and progression [10–12].

However, not all studies support the positive association between sleep disorder and risk of cancer, especially assessing the association between OSAS and specific cancer subtypes [13–15]. Based on a large nationally representative health insurance database, OSAS increased risk of pancreatic and kidney cancer and melanoma, but not colorectal, breast, and prostate cancers [15]. These studies reveal that the association between OSAS and cancer is limited to specific cancer sites or types of malignant cells.

Lung cancer is one of the most frequently diagnosed cancers in the world [16]. The relationship between OSAS and lung cancer has been gained attention in a cohort study, but the authors were unable to find an association between OSAS and lung cancer incidence [15]. Additionally, currently available evidence that assesses the association between OSAS and lung cancer mortality or outcomes is lacking.

To examine these possibilities, we took advantage of existing hospital-based medicine database and gene microarray analyses of datasets from the TCGA and GEO. Firstly, we use FusionInsight HD platform to analyze the relationship between OSAS and lung cancer, and assess the predictive value of OSAS severity that affects cancer outcomes in combination of clinical variables. The FusionInsight HD is an informatics tool that we develop at the First Affiliated Hospital of Zhengzhou University for effective integration of clinical data and providing disease-relevant information to clinical researches. In order to better understand the molecular characteristics, their associated biological function and pathways, we use a public microarray dataset to integrate gene and network-level analyses and evaluate which gene networks are involved in this association of OSAS and lung cancer.

## RESULTS

### Data based on hospital population: OSAS severity was associated with clinical parameters and overall survival in patients with lung cancer

During January 2013 and December 2014, about 500,000 patients admitted in our hospital. According to our inclusion and exclusion criteria, the data of patients with complete information were extracted. The concurrence of lung cancer and OSAS was noted in 43 patients. No significant difference of lung cancer incidence was observed among OSAS patients and hospital population. To analyze the correlation between OSAS and clinical parameters in lung cancer patients, we stratified by OSAS severity categories. Among the co-occurrence of OSAS in patients with lung cancer, 14 had mild OSAS, 11 had moderate OSAS, and 18 had severe OSAS. Tumor size and tumor staging were found to be adherent to OSAS severity category after stratification based on severity of OSAS (measured by AHI). Of the 18 lung cancer patients with severe OSAS, 11/18 (61%) had bigger tumor size, 16/18(88.9%) were in the stage of III/IV. Of the 11 lung cancer patients with moderate OSAS, 5/11(45%) had bigger tumor size, 7/11(64%) were in the stage of III/IV. Of the 14 lung cancer patients with mild OSAS, 1/14 (7%) had bigger tumor size, 2/14(14%) were in the stage of III/IV. No differences in associations were noted when stratified by tumor differentiation, histopathological classification surgery, smoking, drinking categories (Table 1).

**Table 1:** The correlation between apnea degree and clinical parameters of lung cancer in patients with obstructive sleep apnea

Characteristics	N	Obstructive sleep apneasyndrome (OSAS)			χ^2^	P
		Mild	Moderate	Severe		
Sex						
Male	30	9	7	14	0.943	0.624
Female	13	5	4	4		
Age						
<65	25	8	6	11	0.129	0.937
≥65	18	6	5	7		
Pathology						
Adenocarcinoma	34	11	10	13	5.320	0.256
Squamous	6	3	1	2		
Others	3	0	0	3		
Tumor size(T)						
1-2	26	13	6	7	9.812	**0.007**
3-4	17	1	5	11		
Node(N)						
0-1	24	12	5	7	7.645	**0.022**
2-3	19	2	6	11		
Metastasis(M)						
0	24	12	5	7	7.645	**0.022**
1	19	2	6	11		
Stage						
I-II	18	12	4	2	18.193	**0.000**
III-IV	25	2	7	16		
Differentiation						
Well/Moderately	31	7	9	15	5.004	0.080
Poorly	12	7	2	3		
Smoke						
Yes	21	7	5	9	0.068	0.967
No	22	7	6	9		
Drinking						
Yes	10	2	2	6	1.814	0.404
No	33	12	9	12		
Surgery						
Yes	9	5	1	3	2.978	0.226
No	34	9	10	15		

**Figure 1:**
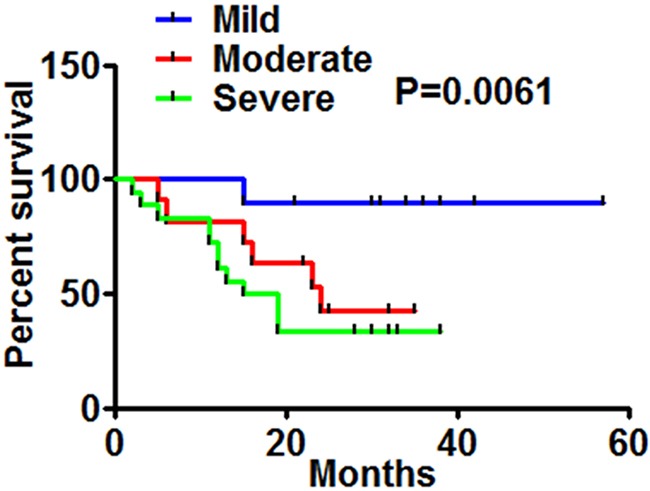
Correlation of OSAS in lung cancer patients and patients’ survival Kaplan-Meier Curves showed association between OSAS severity and patients’ survival in all stages. P values are shown in the graph by log-rank test.

To examine the potential risk for OSAS in cancer patients, we assessed mortality rates for lung cancer in the presence of OSAS. As illustrated in Figure 1, patients with lung cancer who combined with severe or moderate OSAS had a lower overall survival relative to patients with mild OSAS, suggesting that OSAS severity was a risk factor to shorter overall survival in patients with lung cancer.

### Data based on gene cloud of biomedical information: gene network remodeling was involved in lung cancers under hypoxia conditions

**Figure 2:**
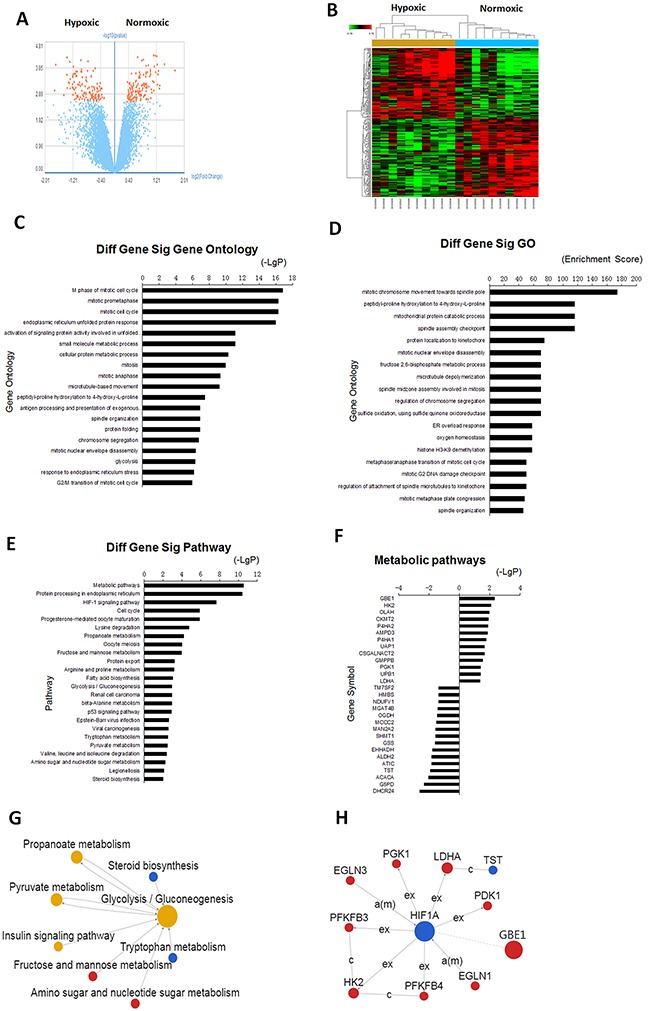
Gene network remodeling was involved in lung cancers under hypoxia conditions **A**. A volcano plot compared gene expression in hypoxic and normoxic fragments of lung cancer. **B**. Graphic display of the gene expression profiles in a heat-map format from hypoxic and normoxic fragments of lung cancer. **C, D, E**. The pathways and gene ontology were significantly affected by hypoxia. **F**. Genes were significantly regulated by hypoxia in metabolic pathways. **G**. Network of gene pathway for differentially expressed transcription factors. **H**. Gene network for the differentially expressed metabolic pathway.

In the gene expression profiling analysis, we examined publicly available human microarray gene expression data (GSE30979) from 10 patients with non-small cell lung cancer. These lung cancer fragments were maintained in culture medium with ambient oxygen or hypoxia (1% O_2_). Previous studies have confirmed that this hypoxic environment resulted in tumor growth and progression. The top 10 up-regulated genes that were differentially expressed between the normoxic group versus the hypoxic group are listed in Supplementary Table 1. Many of the genes enriched in hypoxic-induced lung cancer cells corresponded to tumor invasion and metastasis. For example, lysyl oxidase (LOX) expression is regulated by hypoxia-inducible factor (HIF), which responsible for the invasive properties of human cancer cells through focal adhesion kinase activity and cell to matrix adhesion under hypoxia conditions [17] (Figure 2A–2B, Supplementary Table 2).

To identify significant associations of genes with any specific molecular pathway screened by microarray, we performed annotation enrichment analyses. Genes that significantly enriched for P-value in GO terms included cell cycle, mitotic prometaphase, endoplasmic reticulum unfolded protein response, metabolic process. These biological processes that had high enrichment scores included mitotic chromosome movement towards spindle pole, mitochondrial protein catabolic process, and protein localization to kinetochore (Figure 2C–2D).

Notably, we revealed significant alterations of molecules related to metabolic pathways in the hypoxic-conditioned lung cancer cells that were not previously appreciated by differential expression alone (Supplementary Table 3). This pattern is also reflected in the number of genes in regulated networks, as expected. Many of highly ranked genes in regulated metabolic pathways have also been involved in the top-ranked gene networks, such as glycogen branching enzyme 1(GBE1). Glycolysis/gluconeogenesis is the most enriched in the network of metabolic pathways. Notably, many of these metabolic-related genes have been linked to cancer in the literature, in accord with the multiple connections between cancer and metabolism. In addition to the metabolic pathways enriched within this probe list, other pathways were also significantly regulated by hypoxia, including those involving protein processing in endoplasmic reticulum, and hypoxia-inducible factor (HIF) signaling pathway. Among these pathways, HIF1 signaling is a major adaptive mechanism in tumor growth in a hypoxic microenvironment. Hypoxia and expression of HIF1 are characteristic features of all solid tumors, which involved in angiogenesis, cell survival and treatment resistance [18, 19] (Figure 2E–2F).

Pathway network analysis showed that glycolysis/gluconeogenesis, propanoate metabolism, pyruvate metabolism, insulin pathway, tryptophan metabolism possessed a higher degree in all altered signaling pathways. We also observed that HIF1, GBE1, HK2 were the main central genes, and HIF1 directly interacted with GBE1 and HK2 in the gene network analysis. (Figure 2G–2H).

### Data based on TCGA: GBE1 and HK2 were associated with overall survival in lung cancer patients

**Figure 3:**
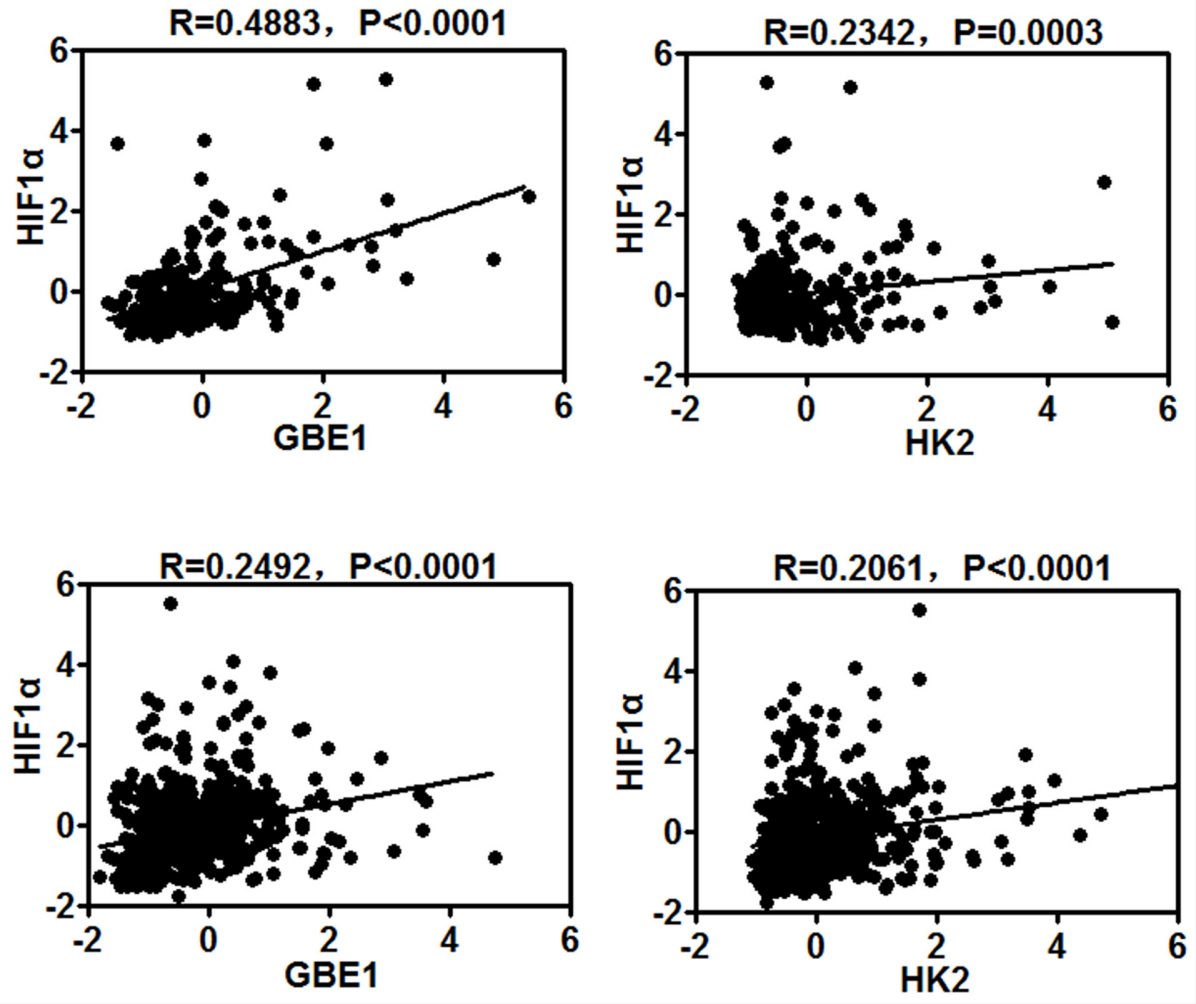
GBE1 and HK2 expression is associated with HIF1α in lung adenocarcinoma and squamous carcinoma patients Scatter plots show correlations of hypoxic-relevant gene (HIF1α) and metabolic specific genes (GBE1, HK2). Black line presents linear interpolation curve between two genes in lung cancer patients. Correlation coefficient R values between two genes were computed using Pearson correlation calculations. All of the P values are shown in the graph.

To characterize if hypoxic-relevant genes are associated with lung cancer, we investigated coexpression patterns of genes in lung cancer. These genes were selected from the following lists: (a) genes believed to play an important role in hypoxia-induced responses, such as HIF1; (b) genes defined as metabolic specific in the literatures and also involved in the top-ranked genes, such as HK2; (c) genes found to be in the top of metabolic pathways, such as GBE1. We constructed gene coexpression networks from gene expression data after adjusting for specific tumor type. We found that GBE1 and HK2 expression pattern has a highly positive correlation with HIF1α in lung adenocarcinoma and squamous carcinoma patients (Figure 3). This result suggested that hypoxic environment functioned as positive regulators of GBE1 and HK2 in lung cancer.

**Figure 4:**
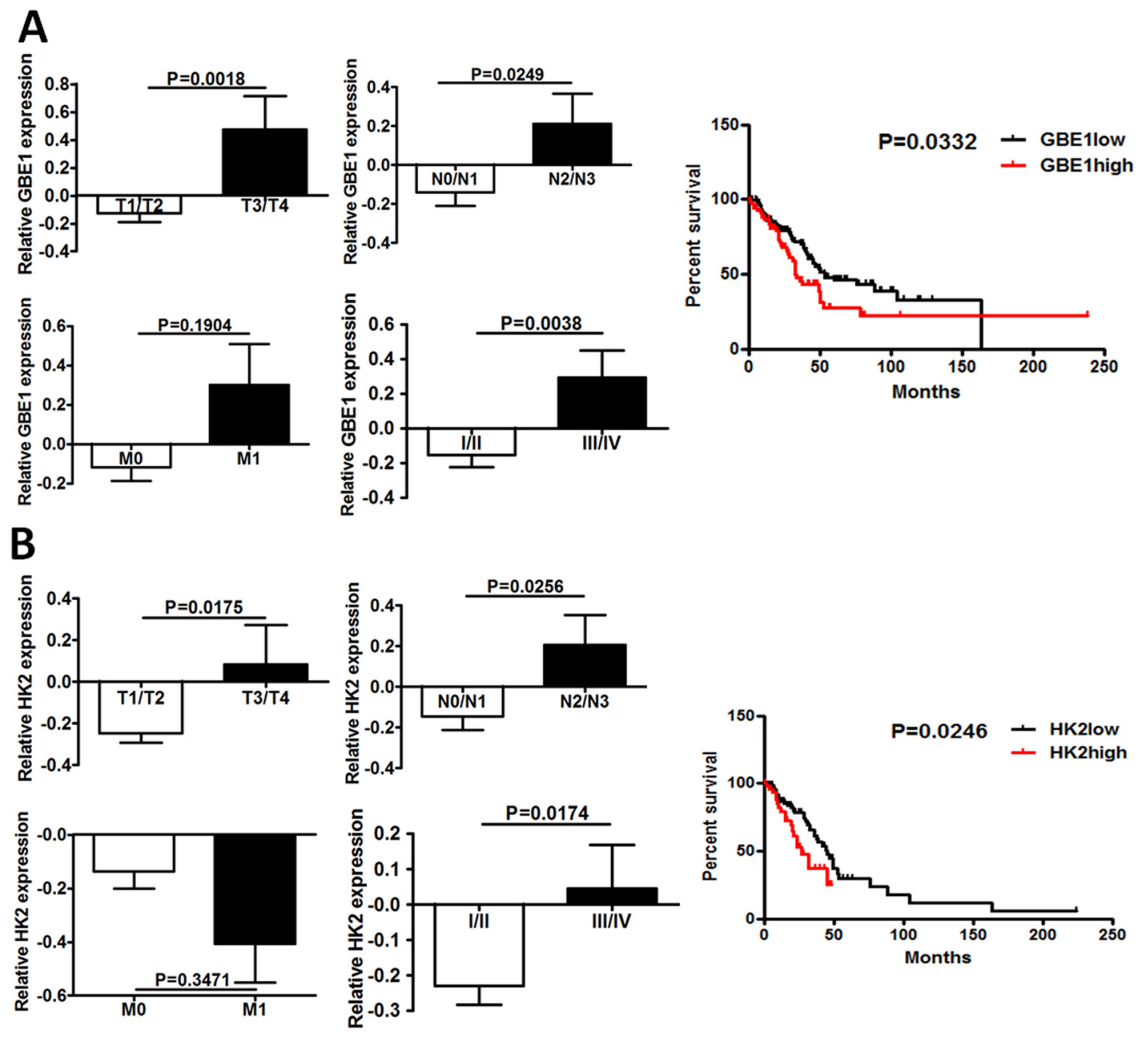
GBE1 and HK2 expression is associated with poor prognosis in lung adenocarcinoma patients The expression of GBE1 and HK2 present in different stages of lung adenocarcinoma patients according to the data retrieved from the TCGA online database. Kaplan-Meier curves show the association between expression of GBE1 and HK2 and overall survival in all stages. All of the P values are shown in the graph.

**Figure 5:**
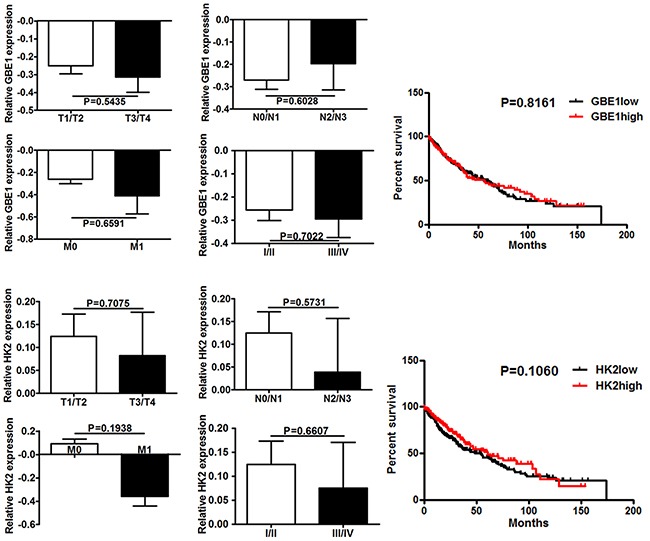
GBE1 and HK2 expression is not associated with prognosis in lung squamous carcinoma patients The expression of GBE1 and HK2 present in different stages of lung squamous carcinoma patients according to the data retrieved from the TCGA online database. Kaplan-Meier curves show the association between expression of GBE1 and HK2 and overall survival in all stages. All of the P values are shown in the graph.

To further assess whether GBE1 and HK2 expression was associated with good or poor survival in lung cancer patients, a publicly available transcriptome dataset was analyzed. A total datasheet included 230 samples with lung adenocarcinoma, and 504 samples with lung squamous cell carcinoma. The patients without clinical events were excluded. We found that in patients with lung adenocarcinoma, expression of HK2 and GBE1 were positively associated with tumor size (P=0.0175, P=0.0018), tumor stage (P=0.00174, P=0.003) and TNM classification N staging (P=0.0256, P=0.0249), respectively. There was no significant correlation between tumor metastasis and gene expression. The survival analysis revealed that in lung adenocarcinoma patients, the ones with high expression of GBE1 and HK2 represented shorter overall survival than those with low expression of GBE1 (P=0.0332) and HK2 (P=0.0246), evidence supporting an association between these genes and cancer survival (Figure 4). When considering the different histological types of lung cancer, no correlations between overall survival and expressions of GBE1 and HK2 were found in squamous carcinoma patients (Figure 5). No significant association between HIF1 expression and survival was found in patients with lung cancer (data no shown).

## DISCUSSION

Our work provides clinical epidemiology data for unraveling the relationship between OSAS and lung cancer, and our analysis have uncovered insights into the molecular mechanisms underlying pathogenesis of OSAS-related comorbidity. Previous studies have addressed there is an association between nocturnal hypoxemia and cancer mortality in patients with OSAS [8, 20–22], while other animal experiments have investigated the deleterious role of intermittent hypoxia in enhancing metastasis and boosting tumor growth [10, 23, 24]. In our work, we integrate clinical data in our hospital to study the risk and clinical outcomes of lung cancer in patients with OSAS. We provide evidence that OSAS is not associated with lung cancer incidence, which is consistent with a longitudinal nationwide-based conclusion [15]. We also confirmed OSAS severity was a risk factor that contributed to short overall survival in patients with lung cancer. Lastly, we found changes of some common pathways such as HIF1 signaling and metabolic pathways are involved in hypoxic-induced lung cancer cells; GBE1 and HK2 as independent unconventional clinical factors predicted survival in lung adenocarcinoma patients based on public microarray datasets. Thus, our results support the notion that the presence of OSAS is deleterious on cancer outcomes; Hypoxia, as one of components of OSAS, is being mechanistically involved in this effects.

Of note, the analysis of relationship between OSAS and lung cancer incidence was based on our hospital database; some inherent limitations of electronic database should be considered. Firstly, we observed that OSAS was not associated with lung cancer based on our hospital data. However, the result must be interpreted with caution owing to the fact that the incidence of lung cancer in OSAS was matched to hospital population-based database, but not a general population-based database or a geographical location to the nationwide level. Also, patients with OSAS were usually diagnosed in respiratory department and neglected in other departments and under mild condition, highlighting the need for further population-based cohort study with OSAS screening and follow-up. In addition, although information on smoking, age, sex, surgery was analyzed in our study, obesity or body mass index, cardiovascular disorders and metabolic abnormalities were not considered, because those data could not be derived from our database. Results from previous studies have provided substantial evidence supporting a bidirectional relationship between metabolic dysfunction and OSAS; the progression of those disorders will be accelerated in the concurrence of OSAS [25, 26], which appears to impose an addition burden on cancer. Accordingly, treatment of OSAS in adult individuals with continuous positive airway pressure lowers blood pressure and partially reverses metabolic abnormalities, suggesting the significant correlation of OSAS and metabolic syndrome [27]. Although concerns were raised regarding the methodologies, our study nevertheless provides the initial recognition and step towards exploration of lung cancer progression in patients with OSAS.

The mechanisms underlying the associations of OSAS and lung cancer have not been explored, but the evidence from animal and cellular models of lung cancer have revealed the relationship between lung cancer and hypoxia. Consistently, our analysis has shown that OSAS is associated with tumor size, lymph node metastasis, tumor stages and survival, but not differentiation. Given this evidence, we argue that hypoxic microenvironment promotes tumor growth in specific lung sites, a conclusion supported by the data that a high-rate intermittent hypoxia mimicking the one experienced by OSAS patients induces melanoma lung metastasis [1]. Other evidence also suggests that hypoxic tumor microenvironment contribute to the tumorigenesis of non-small-cell lung cancer [28]. It should be noted that, no animal or cell models have encompassed the major components of OSAS such as intermittent hypoxia and fragment sleep, and swinging shifts in intrathoracic pressure and episodic hypercapnia [26]. The roles of most of these components have been unexplored in the context of lung cancer. Consequently, the experimental model of hypoxia was used to understand the contribution of OSAS to lung cancer in our analysis.

HIF1 is a master transcriptional regulator in the response of hypoxic, which balance oxygen supply and demand. HIF1-associated transcriptional network contributes to angiogenesis, stress oxidative response, immune evasion, invasion and metastasis, metabolic reprogramming in the tumor pathogenesis [29]. Our analysis confirmed HIF1 signaling implicated in the lung cancer progression in patients with OSAS, which is consistent with previous work showing that HIF1 specifically increased lung cell adhesion, clotting, and fibrin deposition [30, 31]. In addition, our analysis also showed that distinguished gene signature regulated by hypoxia primarily consisted of metabolic-specific genes, in particular genes involved in glycolysis and gluconeogenesis. This result indicated that HIF1-meidated metabolic pathways particularly contribute lung cancer under conditions of hypoxia. That is because HIF1 can directly bind the promoters of those genes of glycolytic enzymes and glucose transporters, which promote the shift from oxidative to glycolytic metabolism and reduce the oxygen consumption [32]. We note that previous experiments also focus on other consequences of HIF activation such as HIF1-dependent upregulation of vascular endothelial growth factor (VEGF), which can improve the supply of oxygen and implicate in the tumor progression. However, VEGF-related network did not significantly change in our analysis. Thus, it is intriguing that HIF may exert its deleterious effects through specific metabolic pathway in patients with concurrent presence of OSAS and cancer, but further work needs to be done to disentangle these complex HIF-related networks in lung cancer.

We mapped metabolic network, and showed that expressions of GBE1 and HK2 significantly upregulated and highly correlated with expression of HIF1 in lung cancer. Furthermore, a significant association emerged between expressions of GBE1/HK2 and the lung adenocarcinoma, but not lung squamous carcinoma in our analysis. This difference may be explained partly by different clinical and pathologic features of these two type lung cancer. Squamous cell carcinoma mostly develops in smokers, in whom lymph node metastases or vascular invasion often develop [33, 34]. HIF1 up-regulates expression of HK2 by binding to hypoxia-responsive elements (HREs) in the HK2 promoter. Tumors with decreased HK2 expression showed alterations in VEGF-A signaling, a pathway important for angiogenesis and metastasis [35]. In lung cancer cell lines, HK2 was required for the human and mouse lung cancer cell growth; inhibition of HK2 inhibited human and mouse lung cancer cell growth through inducing cell apoptosis and autophagy [36]. Other evidence also suggests that HIF1 regulates GBE1 expression, which is involved in the biosynthesis of glycogen [37, 38]. Our work presents evidence that alteration of HIF1 in lung cancer cells is associated with HK2 and GBE1.

Finally, ascertaining whether there is a direct relationship between cancer incidence or progression or mortality and the severity of OSAS, would require future studies on cohorts with a high number of patients over an extended time period. Well-controlled conditions to investigate the effect of intermittent hypoxia in animal models will provide direct support for a hypothesis of a causal link between OSAS and cancer. The convergence of HIF1 and HK2/GBE1 in the lung cancer patients with OSAS raises important questions regarding the underlying mechanism of OSAS-lung cancers. Nonetheless, our analysis provides clinical implications for the evaluation and treatment of OSAS in lung cancers.

## MATERIALS AND METHODS

### Clinical data source and study population

The data request and data extraction underwent by using FusionInsight HD platform, which based on framework of Hadoop big data mining technology [39, 40, 41]. We retrospectively reviewed the electronic medical records system of all patients who admitted to our hospital from 1 January 2013 to 31 December 2014. Consecutive patients >18 years who had been assessed for OSAS and lung cancer were eligible. For the patients with concurrence of lung cancer and OSAS, those with continuous positive airway pressure treatment were excluded; those with multiple cancers or metastasis to the lungs were excluded.

Obstructive sleep apnea is defined as an apnea–hypopnea index (AHI) score of 5 or more events per hour of sleep. The severity of OSAS is rated as mild (AHI 5 to <15), moderate (AHI 15 to<30), or severe (AHI>30). The lung cancer patients with diagnosis of OSAS were registered and scheduled follow-up.

Patients and clinical characteristics included basic demographics (age, sex, drinking, smoking), comorbidities, malignancy (region of cancer, pathology, disease status, treatment), sleep history (previous diagnosis of OSAS, sleep symptoms), physical examination, sleep stage, apneas, hypopneas, oxygen saturation nadir, positive pressure therapy), and treatment of sleep disorder. We used Kaplan-Meier curves to present the prognosis of lung cancer plus OSAS patients with mild-, moderate- and severe-degree groups.

### Microarray gene expression data collection include GO and pathway analysis regarding the association of OSAS and lung cancer

Intermittent hypoxia and sleep fragmentation are two major components of OSAS; both of them have been implicated in tumorigenesis. However, since no public human microarray gene expression data regarding the association of sleep fragmentation and lung cancer are available now, here we only analysis one factor of OSAS—hypoxia in the pathogenesis of lung cancer. Katharina Leithner et al. (2014) studied differential gene expression profiles in human lung cancer tissue under different oxygen microenvironment using Affymetrix Gene Chip 1.0 ST microarrays. Non-small cell lung cancer fragments were maintained ex vivo in normoxia or hypoxia condition. Viability, apoptosis rates and tissue hypoxia are used to access responses to hypoxia. The dataset consists of 10 normoxia samples and 10 hypoxia samples. The microarray data are available at Gene Expression Omnibus (GEO:http://www.ncbi.nlm.nih.gov/geo/; accession number: GSE30979).

Gene expression data (GSE30979 profiling data) were downloaded as raw signals from Gene Expression Omnibus (http://www.ncbi.nlm.nih.gov/geo), interpreted, normalized and log2 scaled using the online analysis tool: Gene Cloud of Biomedical information (GCBI) website (https://www.gcbi.com.cn). Exploring of differentially expressed gene sets between normal and cancer stromal profiles in GSE30979 was also performed via the GCBI online tool. This is a public database containing information from chip and sequencing analysis that permits to investigate the association of genes with differentially expressed intersection mRNAs were entered into the Gene Ontology database (http://www.geneontology.org), which utilized GO to identify the molecular function represented in the gene profile. Up and down regulated genes were analyzed, respectively. The Kyoto Encyclopedia of Genes and Genomes (KEGG) (http://www.kegg.jp/) was used to analyze the potential functions of these genes participated in the pathways.

### Clinical features and survival analysis

The mRNA expression z-Scores (RNA Seq V2 RSEM) of GBE1 and HK2 in lung adenocarcinoma and squamous cell carcinoma was performed using cBioPortal for Cancer Genomics (http://www.cbioportal.org). All searches were performed according to the cBioPortal's online instructions. The database query was based on mRNA expression of the GBE1 and HK2 in TCGA lung adenocarcinoma(TCGA, Nature 2014) and lung squamous cell carcinoma (TCGA, Provisional), respectively. A total of 230 samples with lung adenocarcinoma, and 504 samples with lung squamous cell carcinoma had clinical and survival information (downloaded 05/20/2016). Patients without clinical events during the study were considered censored. Clinical events include molecular features originally defined in tumor tissues, survival and patients status related features, such as histology and tumor weight.

Overall survival analysis of the patient with high and low levels of GBE1 and HK2 based on TCGA RNA-Seq data set was shown by using a Kaplan-Meier survival plot. We used Kaplan-Meier curves to present the prognosis of the higher- and lower-risk groups. The Wilcoxon log-rank test was then conducted on the Kaplan-Meier curves to detect the survival difference between these two groups. All survival analysis was conducted using the R package Survival.

### Statistical analysis

Based on the distribution level, data are reported as mean ± SD and correlation analysis were evaluated with parametric (Independent-Sample or paired T test and Spearman's test) or non-parametric (Wilcoxon and Spearman's ρ test) tests. A value of P < 0.05 was considered statistically significant. Significant correlations were determined using 2-tailed (or 1-tailed where designated) Pearson correlation calculations. All statistics and graph preparations were performed using Prism 6 (Graph Pad Software Inc.). Statistical analysis of significance was calculated by one-way analysis of variance followed by Tukey's post hoc test with SPSS 16.0 for Windows (SPSS, Chicago, IL).

## SUPPLEMENTARY MATERIALS TABLES



## References

[1] Almendros I, Montserrat JM, Torres M, Dalmases M, Cabanas ML, Campos-Rodriguez F, Navajas D, Farre R (2013). Intermittent hypoxia increases melanoma metastasis to the lung in a mouse model of sleep apnea. Respir Physiol Neurobiol.

[2] Peppard PE, Young T, Palta M, Skatrud J (2000). Prospective study of the association between sleep-disordered breathing and hypertension. N Engl J Med.

[3] Loke YK, Brown JW, Kwok CS, Niruban A, Myint PK (2012). Association of obstructive sleep apnea with risk of serious cardiovascular events: a systematic review and meta-analysis. Circ Cardiovasc Qual Outcomes.

[4] Howard ME, Desai AV, Grunstein RR, Hukins C, Armstrong JG, Joffe D, Swann P, Campbell DA, Pierce RJ (2004). Sleepiness, sleep-disordered breathing, and accident risk factors in commercial vehicle drivers. Am J Respir Crit Care Med.

[5] Babu AR, Herdegen J, Fogelfeld L, Shott S, Mazzone T (2005). Type 2 diabetes, glycemic control, and continuous positive airway pressure in obstructive sleep apnea. Arch Intern Med.

[6] Chung SA, Yuan H, Chung F (2008). A systemic review of obstructive sleep apnea and its implications for anesthesiologists. Anesth Analg.

[7] Peppard PE, Nieto FJ (2013). Here come the sleep apnea-cancer studies. Sleep.

[8] Nieto FJ, Peppard PE, Young T, Finn L, Hla KM, Farre R (2012). Sleep-disordered breathing and cancer mortality: results from the Wisconsin Sleep Cohort Study. Am J Respir Crit Care Med.

[9] Chang WP, Liu ME, Chang WC, Yang AC, Ku YC, Pai JT, Lin YW, Tsai SJ (2014). Sleep apnea and the subsequent risk of breast cancer in women: a nationwide population-based cohort study. Sleep Med.

[10] Almendros I, Wang Y, Becker L, Lennon FE, Zheng J, Coats BR, Schoenfelt KS, Carreras A, Hakim F, Zhang SX, Farre R, Gozal D (2014). Intermittent hypoxia-induced changes in tumor-associated macrophages and tumor malignancy in a mouse model of sleep apnea. Am J Respir Crit Care Med.

[11] Hakim F, Wang Y, Zhang SX, Zheng J, Yolcu ES, Carreras A, Khalyfa A, Shirwan H, Almendros I, Gozal D (2014). Fragmented sleep accelerates tumor growth and progression through recruitment of tumor-associated macrophages and TLR4 signaling. Cancer Res.

[12] Khalyfa A, Almendros I, Gileles-Hillel A, Akbarpour M, Trzepizur W, Mokhlesi B, Huang L, Andrade J, Farre R, Gozal D (2016). Circulating exosomes potentiate tumor malignant properties in a mouse model of chronic sleep fragmentation. Oncotarget.

[13] Christensen AS, Clark A, Salo P, Nymann P, Lange P, Prescott E, Rod NH (2013). Symptoms of sleep disordered breathing and risk of cancer: a prospective cohort study. Sleep.

[14] Zhang XB, Peng LH, Lyu Z, Jiang XT, Du YP (2015). Obstructive sleep apnoea and the incidence and mortality of cancer: a meta-analysis. Eur J Cancer Care (Engl).

[15] Gozal D, Ham SA, Mokhlesi B (2016). Sleep Apnea and Cancer: Analysis of a Nationwide Population Sample. Sleep.

[16] Pineros M, Sierra MS, Forman D (2016). Descriptive epidemiology of lung cancer and current status of tobacco control measures in Central and South America. Cancer Epidemiol.

[17] Erler JT, Bennewith KL, Nicolau M, Dornhofer N, Kong C, Le QT, Chi JT, Jeffrey SS, Giaccia AJ (2006). Lysyl oxidase is essential for hypoxia-induced metastasis. Nature.

[18] Triner D, Shah YM (2016). Hypoxia-Inducible Factors: a central link between inflammation and cancer. J Clin Invest.

[19] Semenza GL (2013). HIF-1 mediates metabolic responses to intratumoral hypoxia and oncogenic mutations. J Clin Invest.

[20] Campos-Rodriguez F, Martinez-Garcia MA, Martinez M, Duran-Cantolla J, L Pena Mde, Masdeu MJ, Gonzalez M, Campo F, Gallego I, Marin JM, Barbe F, Montserrat JM, Farre R (2013). Association between obstructive sleep apnea and cancer incidence in a large multicenter Spanish cohort. Am J Respir Crit Care Med.

[21] Martinez-Garcia MA, Campos-Rodriguez F, Duran-Cantolla J, M de la Pena, Masdeu MJ, Gonzalez M, F Del Campo, Serra PC, Valero-Sanchez I, Ferrer MJ, Marin JM, Barbe F, Martinez M (2014). Obstructive sleep apnea is associated with cancer mortality in younger patients. Sleep Med.

[22] Fang HF, Miao NF, Chen CD, Sithole T, Chung MH (2015). Risk of Cancer in Patients with Insomnia, Parasomnia, and Obstructive Sleep Apnea: A Nationwide Nested Case-Control Study. J Cancer.

[23] Almendros I, Montserrat JM, Ramirez J, Torres M, Duran-Cantolla J, Navajas D, Farre R (2012). Intermittent hypoxia enhances cancer progression in a mouse model of sleep apnoea. Eur Respir J.

[24] Cairns RA, Kalliomaki T, Hill RP (2001). Acute (cyclic) hypoxia enhances spontaneous metastasis of KHT murine tumors. Cancer Res.

[25] Basner RC (2014). Cardiovascular morbidity and obstructive sleep apnea. N Engl J Med.

[26] Gileles-Hillel A, Kheirandish-Gozal L, Gozal D (2016). Biological plausibility linking sleep apnoea and metabolic dysfunction. Nat Rev Endocrinol.

[27] Sharma SK, Agrawal S, Damodaran D, Sreenivas V, Kadhiravan T, Lakshmy R, Jagia P, Kumar A (2011). CPAP for the metabolic syndrome in patients with obstructive sleep apnea. N Engl J Med.

[28] Foster JG, Wong SC, Sharp TV (2014). The hypoxic tumor microenvironment: driving the tumorigenesis of non-small-cell lung cancer. Future Oncol.

[29] Mockler MB, Conroy MJ, Lysaght J (2014). Targeting T cell immunometabolism for cancer immunotherapy; understanding the impact of the tumor microenvironment. Front Oncol.

[30] Nakajima T, Anayama T, Koike T, Shingyoji M, Castle L, Kimura H, Yoshino I, Yasufuku K (2012). Endobronchial ultrasound doppler image features correlate with mRNA expression of HIF1-alpha and VEGF-C in patients with non-small-cell lung cancer. J Thorac Oncol.

[31] Evans CE, Bendahl PO, Belting M, Branco C, Johnson RS (2016). Diverse roles of cell-specific hypoxia-inducible factor 1 in cancer-associated hypercoagulation. Blood.

[32] Loftus RM, Finlay DK (2016). Immunometabolism: Cellular Metabolism Turns Immune Regulator. J Biol Chem.

[33] Usui S, Minami Y, Shiozawa T, Iyama S, Satomi K, Sakashita S, Sato Y, Noguchi M (2013). Differences in the prognostic implications of vascular invasion between lung adenocarcinoma and squamous cell carcinoma. Lung Cancer.

[34] Kawase A, Yoshida J, Ishii G, Nakao M, Aokage K, Hishida T, Nishimura M, Nagai K (2012). Differences between squamous cell carcinoma and adenocarcinoma of the lung: are adenocarcinoma and squamous cell carcinoma prognostically equal?. Jpn J Clin Oncol.

[35] Anderson M, Marayati R, Moffitt R, Yeh JJ (2016). Hexokinase 2 promotes tumor growth and metastasis by regulating lactate production in pancreatic cancer. Oncotarget.

[36] Wang H, Wang L, Zhang Y, Wang J, Deng Y, Lin D (2016). Inhibition of glycolytic enzyme hexokinase II (HK2) suppresses lung tumor growth. Cancer Cell Int.

[37] Zhao J, Chen H, Davidson T, Kluz T, Zhang Q, Costa M (2004). Nickel-induced 1,4-alpha-glucan branching enzyme 1 up-regulation via the hypoxic signaling pathway. Toxicol Appl Pharmacol.

[38] Pescador N, Villar D, Cifuentes D, Garcia-Rocha M, Ortiz-Barahona A, Vazquez S, Ordonez A, Cuevas Y, Saez-Morales D, Garcia-Bermejo ML, Landazuri MO, Guinovart J, del Peso L (2010). Hypoxia promotes glycogen accumulation through hypoxia inducible factor (HIF)-mediated induction of glycogen synthase 1. PLoS One.

[39] Yao Q, Tian Y, Li PF, Tian LL, Qian YM, Li JS (2015). Design and development of a medical big data processing system based on Hadoop. J Med Syst.

[40] Jayapandian CP, Chen CH, Bozorgi A, Lhatoo SD, Zhang GQ, Sahoo SS Cloudwave: distributed processing of "big data" from electrophysiological recordings for epilepsy clinical research using Hadoop. AMIA Annu Symp Proc.

[41] Fang Z, Fan X, Chen G (2014). A study on specialist or special disease clinics based on big data. Front Med.

