# Supplementary material for: Target of obstructive sleep apnea syndrome merge lung cancer: based on big data platform
Source: Oncotarget. 2017 Feb 16;8(13):21567–78. doi: 10.18632/oncotarget.15372 (PMC5400607; doi:10.18632/oncotarget.15372)
Supplement: Supplementary file 1 [file oncotarget-08-21567-s001.pdf]

Target of obstructive sleep apnea syndrome merge lung cancer: based on big data platform

SUPPLEMENTARY TABLES

Supplementary Table 1: Top 10 hypoxia up-regulated genes according to fold change

Gene	Fold change	P-value	Gene ontology (Biological process)	Gene feature	Lung cancer articles
MROH9	3.35	0.000177		Up	0
LOX	2.7	0.000104	oxidation reduction/lung development	Up	16
FICD	2.4	0.000827	protein adenylyltransferase activity	Up	0
NUCB2	2.38	0.000184		Up	1
DNAJB9	2.35	0.000243	misfolded protein binding	Up	0
HILPDA	2.35	0.000106	autocrine signalingregulation of cytokine regulation of cell proliferation regulation of lipid deposition	Up	0
GBE1	2.32	0.00059	small-molecule compound metabolism /glucose metabolism/ carbohydrate metabolism	Up	0
IL8	2.27	0.000052	cytokine mediated signal pathway	Up	0
FAM115C	2.25	0.000204	regulation of targeting signals in membrane positive regulation of cell migration negative regulation of anion channel transportation hematopoietic progenitor cell differentiation	Up	0
KCTD11	2.21	0.00005	signaling pathways in smooth muscle neurons differentiation growth regulationu biquitinated protein negative regulation of nerve cell proliferation Cell cycle	Up	0
HSPA13	2.19	0.000479		Up	0

Supplementary Table 2: Top 10 hypoxia-regulated pathways according to p-values

Pathway name	Diff gene counts in pathway	Gene amount in pathway	Enrichment Score	p-value	False discovery rate	Rank	-log(P)
Metabolic pathways	30	1189	4.3923465	3.00E-11	2.76E-09	1	34.956235
Protein processing in endoplasmic reticulum	13	167	13.551397	3.88E-11	2.76E-09	2	34.584496
HIF-1 signaling pathway	9	106	14.78066	2.39E-08	1.13E-06	3	25.319933
Cell cycle	8	124	11.231183	1.32E-06	3.94E-05	4	19.535752
Progesterone-mediated oocyte maturation	7	86	14.169574	1.39E-06	3.94E-05	5	19.459992
Lysine degradation	5	49	17.763605	1.86E-05	0.0004414	6	15.710473
Propanoate metabolism	4	32	21.760417	6.74E-05	0.0013667	7	13.85744
Oocyte meiosis	6	112	9.3258929	9.74E-05	0.0017102	8	13.325648
Fructose and mannose metabolism	4	36	19.342593	0.0001084	0.0017102	9	13.171469
Protein export	3	23	22.706522	0.0006093	0.008537	10	10.680474

Supplementary Table 3: Genes were up-regulated by hypoxia in metabolic pathways

Gene symbol	Gene description	d Score	Fold change	p-value	Lung cancer articles
GBE1	"Homo sapiens glucan (1,4-alpha-), branching enzyme 1 (GBE1), mRNA."	4.535219	2.320145	0.000052	0
P4HA2	cdna:known chromosome:GRCh37:5:131527531:131563508:-1 gene:ENSG00000072682 gene_biotype:protein_codingtranscript_biotype:protein_coding	3.592208	1.879895	0.000119	1
AMPD3	"Homo sapiens adenosine monophosphate deaminase 3 (AMPD3), transcript variant 1, mRNA."	3.176169	1.861438	0.000296	0
CSGALNACT2	"Homo sapiens chondroitin sulfate N-acetylgalactosaminyltransferase 2 (CSGALNACT2), mRNA."	3.144939	1.659166	0.000323	0
GMPPB	"Homo sapiens GDP-mannose pyrophosphorylase B (GMPPB), transcript variant 1, mRNA."	3.286918	1.529535	0.000224	0
CKMT2	"Homo sapiens creatine kinase, mitochondrial 2 (sarcomeric) (CKMT2), nuclear gene encoding mitochondrial protein, transcript variant 1, mRNA."	2.904499	1.90858	0.000653	0
P4HA1	"Homo sapiens prolyl 4-hydroxylase, alpha polypeptide I (P4HA1), transcript variant 2, mRNA."	2.93222	1.769448	0.000604	0
UPB1	"Homo sapiens ureidopropionase, beta (UPB1), mRNA."	2.92749	1.384235	0.000612	0
HK2	"Homo sapiens hexokinase 2 (HK2), mRNA."	2.712775	2.101783	0.001147	3
PGK1	"Homo sapiens phosphoglycerate kinase 1 (PGK1), mRNA."	2.678761	1.400536	0.001275	2
UAP1	"Homo sapiens UDP-N-acteylglucosaminepyrophosphorylase 1 (UAP1), mRNA."	2.653412	1.6666	0.001376	0
OLAH	"Homo sapiens oleoyl-ACP hydrolase (OLAH), transcript variant 1, mRNA."	2.520686	1.959926	0.002075	0
LDHA	"Homo sapiens lactate dehydrogenase A (LDHA), transcript variant 1, mRNA."	2.466087	1.37253	0.002455	8
